# Innovative Management of Gummy Smile With Direct Composite Veneers Guided by the Crescent Metal Matrix: A Clinical Case

**DOI:** 10.7759/cureus.96371

**Published:** 2025-11-08

**Authors:** Majed Amran, Aziz Abdullah, Ali A Abdullah, Mohammad Abdullah

**Affiliations:** 1 Department of Endodontics and Operative Dentistry, Tishreen university, Lattakia, SYR; 2 Department of Endodontics and Operative Dentistry, Tishreen University, Lattakia, SYR; 3 Department of Oral and Maxillofacial Surgery, Tishreen University, Lattakia, SYR

**Keywords:** composite, crescent metal matrix, gingival smile, gummy smile, veneer

## Abstract

A gingival (gummy) smile is a common esthetic concern that can affect facial harmony and patient confidence. Its etiology may involve altered passive eruption, hyperactive lip musculature, vertical maxillary excess, or dentoalveolar factors, making accurate diagnosis essential for successful management. This case report presents a minimally invasive approach for gingival smile correction and anterior esthetic rehabilitation using a novel pre-contoured metal matrix system in combination with direct composite restorations.

A 28-year-old patient presented with excessive gingival display and small anterior teeth, compounded by existing crowns on the maxillary central incisors. Treatment involved precise gingival trimming guided by the metal matrix to achieve optimal contour and symmetry, followed by the restoration of teeth #11 and #21 with customized composite crowns reinforced with a fiber post. Composite veneers were then applied to enhance overall esthetics. The procedure was complemented by topical application of a corticosteroid oral paste to promote healing and comfort.

At three-month and subsequent follow-ups, the gingival tissues exhibited healthy contour and stability, and the restorations demonstrated excellent esthetic integration and function. The described approach highlights the effectiveness of integrating innovative matrix-guided contouring with conservative restorative techniques to achieve predictable esthetic and periodontal outcomes in the management of a gingival smile.

## Introduction

A gingival smile, commonly known as a gummy smile, is characterized by the excessive display of gingival tissue, typically exceeding 3 to 4 mm, during smiling. Although it is not considered a pathological condition, this esthetic concern can significantly impact a patient’s self-esteem and psychosocial well-being [1]. The etiology of a gingival smile is multifactorial, including altered passive eruption, vertical maxillary excess, hyperactive upper lip musculature, and dentoalveolar extrusion. Accurate diagnosis is essential, as treatment strategies must be tailored to the underlying cause [2].

Treatment modalities vary accordingly: esthetic crown lengthening or gingivectomy is indicated for altered passive eruption; orthognathic surgery addresses skeletal discrepancies; botulinum toxin (Botox) injections manage hyperactive lip musculature; and orthodontic intrusion corrects dentoalveolar causes. A multidisciplinary approach involving periodontics, orthodontics, and, when necessary, oral surgery is often required to achieve optimal outcomes [3].

Recent advances in soft-tissue management emphasize the importance of gingival phenotype modification in achieving long-term esthetic and periodontal stability. Injectable platelet-rich fibrin (i-PRF) has demonstrated potential in enhancing gingival thickness and resilience, thereby improving clinical outcomes in minimally invasive esthetic procedures [4].

The gingival phenotype plays a critical role in determining esthetic predictability and tissue stability following surgical or restorative interventions. A thin phenotype, in particular, is associated with a higher risk of marginal recession and compromised healing. Recent studies have proposed targeted interventions to enhance soft-tissue thickness and resilience, thereby improving long-term outcomes in esthetic procedures [5].

The Crescent Metal Matrix is a specialized dental matrix system designed to enhance precision and esthetics in anterior restorative procedures. Its distinctive crescent-shaped, pre-contoured metal design closely follows the natural curvature of anterior teeth, allowing for superior adaptation and ease of use. Initially developed to overcome challenges in anterior restorations, this matrix system is particularly effective in Class V restorations and composite veneers, where achieving ideal contour and gingival adaptation is critical. The thin, flexible metal facilitates accurate anatomical form and emergence profile, rendering it an invaluable tool for clinicians striving for high-quality esthetic results. Clinical data have also shown that matrix-guided anterior restorations can achieve superior contour accuracy and esthetic harmony compared to freehand composite sculpting, particularly in ultrathin veneer applications [6].

Gingival trimming using a soft tissue trimmer represents a minimally invasive technique employed to contour or remove excess gingival tissue with precision and minimal patient discomfort. The soft tissue trimmer, typically a rotary instrument equipped with a specialized tip, operates at high speeds and simultaneously coagulates and excises soft tissue, thereby reducing bleeding. This instrument is commonly used for gingival recontouring, exposing subgingival margins, and refining cervical outlines prior to composite veneer placement. Compared to traditional scalpel or electrosurgical techniques, the soft tissue trimmer offers enhanced control, accelerated healing, and improved patient comfort, making it a preferred tool in esthetic and restorative dentistry [7].

## Case presentation

**Figure 1:**
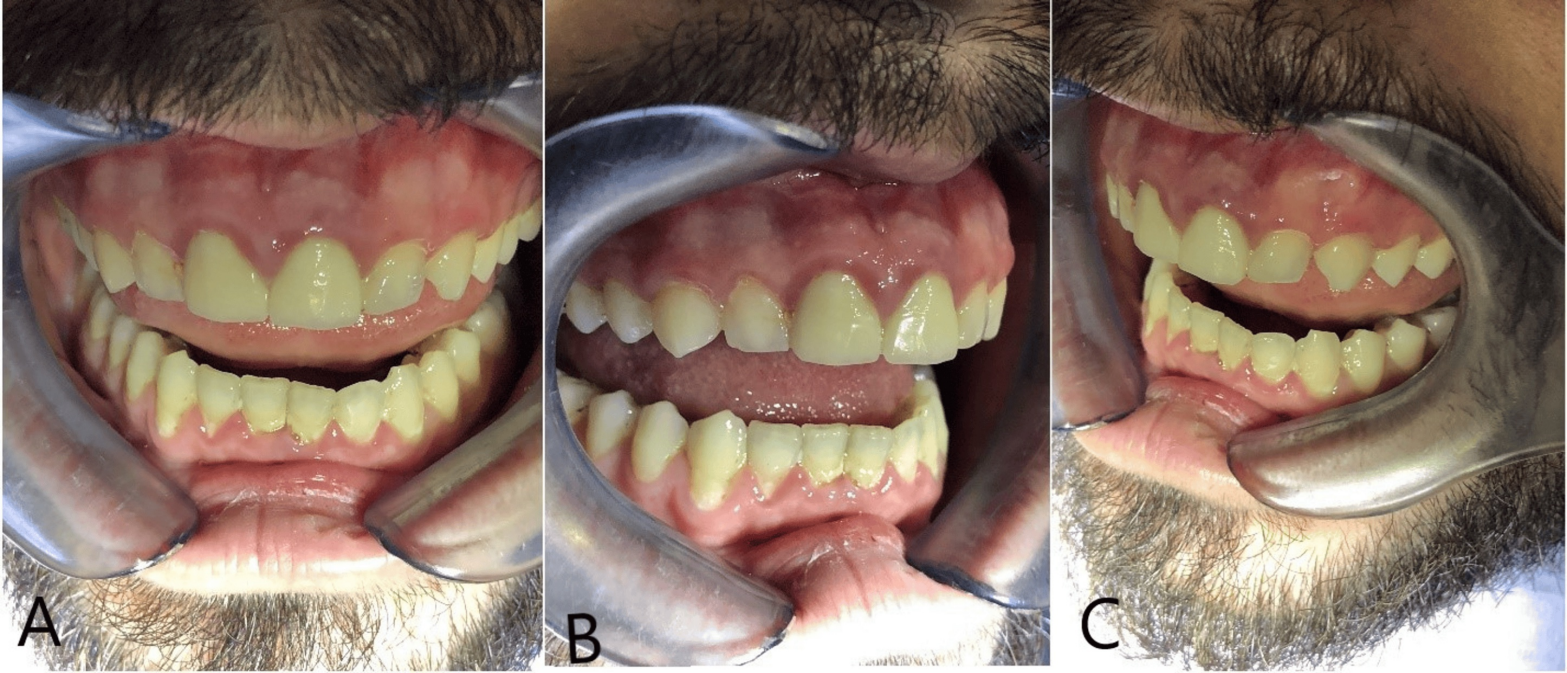
The patient’s small-sized teeth and signs of gingivitis from three different angles: frontal and two lateral views Clinical images showing the patient’s small-sized anterior teeth and gingival inflammation. Views include (A) frontal, (B) right lateral, and (C) left lateral perspectives.

A 28-year-old patient presented to the clinic with the chief complaint of an excessive gingival display during smiling (gingival smile) and concerns about the small size of their anterior teeth. The patient also reported occasional gum bleeding and sensitivity, consistent with signs of gingivitis. The patient met the inclusion criteria for anterior esthetic rehabilitation and gingival recontouring. Written informed consent was obtained for both the clinical treatment and publication of case details and images. Preoperative periodontal therapy, including oral hygiene instruction and scaling, was performed to optimize gingival health prior to the procedure. Clinical examination revealed generalized mild gingival inflammation characterized by redness and bleeding on probing. Notably, teeth #11 and #21 were restored with crowns, which showed no signs of failure but contributed to the overall esthetic imbalance due to their size and contour (Figure 1).

The patient expressed a desire for esthetic rehabilitation to address both the gummy smile and the appearance of small teeth, with particular focus on improving the anterior maxillary esthetics.

Note: Quantitative periodontal indices (e.g., gingival display in mm, probing depths, plaque/bleeding scores) were not recorded at baseline due to the clinical nature of the setting. Assessment of esthetic improvement and tissue stability is based on photographic documentation and clinical observation.

Treatment plan and procedure

**Figure 2:**
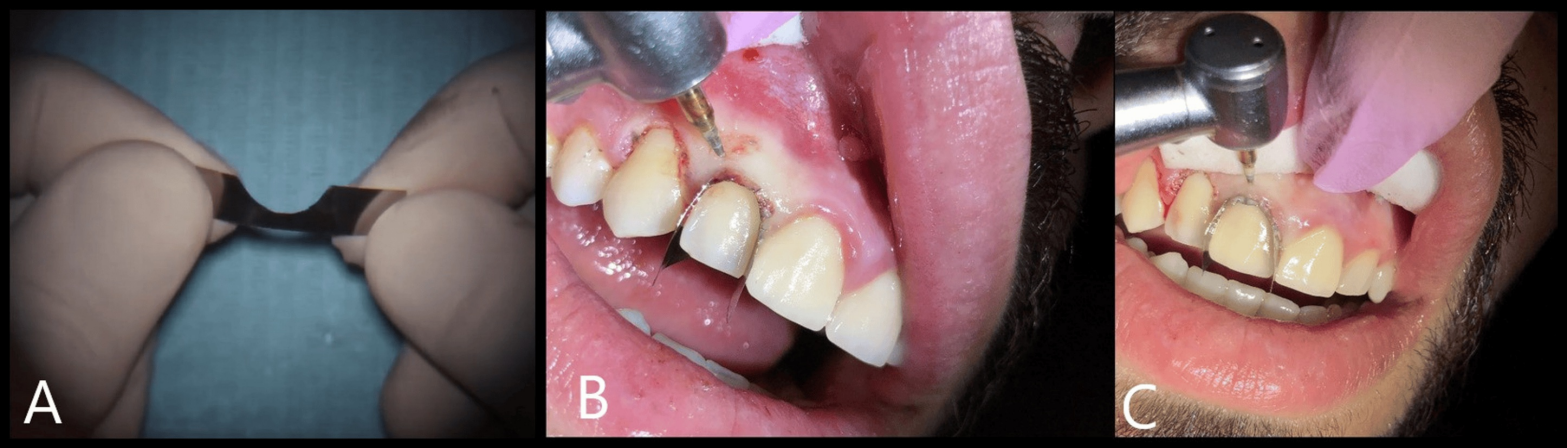
The crescent metal matrix technique and its application in achieving precise contouring and guidance during gingival trimming A. The mesiodistal tooth width is initially estimated from the buccal aspect. Subsequently, a segment of the Tofflemire matrix band is meticulously trimmed to adapt to the tooth’s anatomical contours, spanning from the mesial to the distal surface (or vice versa). Thereafter, a crescent-shaped portion of the band is excised, creating a space that precisely corresponds to the intended depth of the gingival sulcus. B. Application of the crescent metal matrix on tooth #12, with the crescent-shaped edge positioned toward the incisal margin, to guide gingival trimming using the soft tissue trimmer. C. To initiate gingival trimming on tooth #11, a new matrix was applied following the gingival trimming of tooth #12.

After a thorough examination, the treatment plan was discussed in detail with the patient, focusing on addressing the excessive gingival display and improving the esthetics of the anterior teeth. To achieve precise gingival contouring, the Crescent Metal Matrix was employed as a novel tool to aid in gingival retraction and guide the trimming process. This matrix facilitated tooth-by-tooth gingival trimming, allowing controlled removal of excess soft tissue while carefully preserving the biological width (Figure 2).

**Figure 3:**
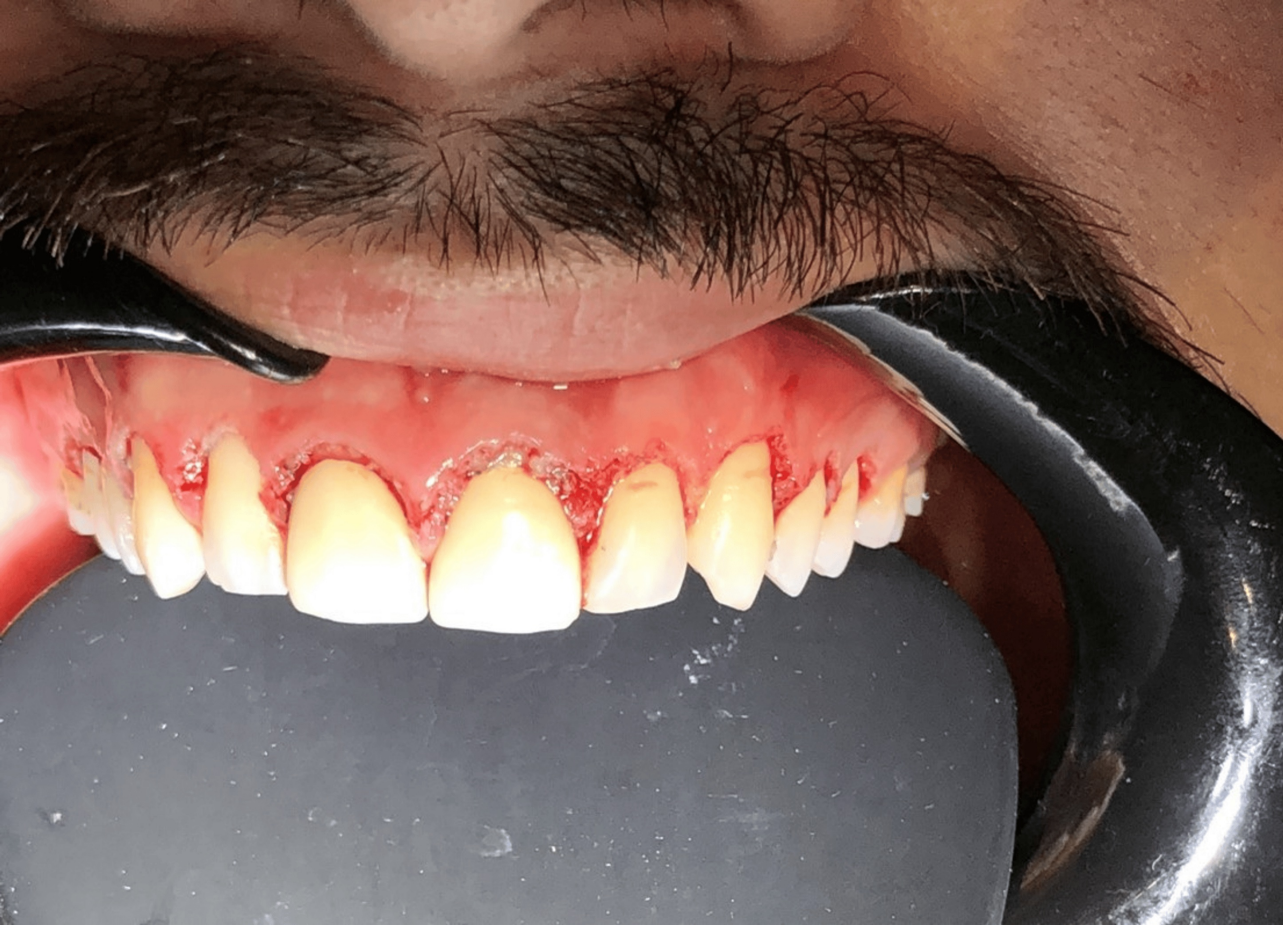
The gingiva immediately after trimming The gingiva immediately after trimming using a soft tissue trimmer and crescent metal matrix, showing clean and well-defined gingival margins

By providing a clear and stable reference for the ideal gingival margin, the matrix ensured that only the appropriate amount of gingiva was removed, optimizing both esthetic outcomes and periodontal health (Figure 3).

**Figure 4:**
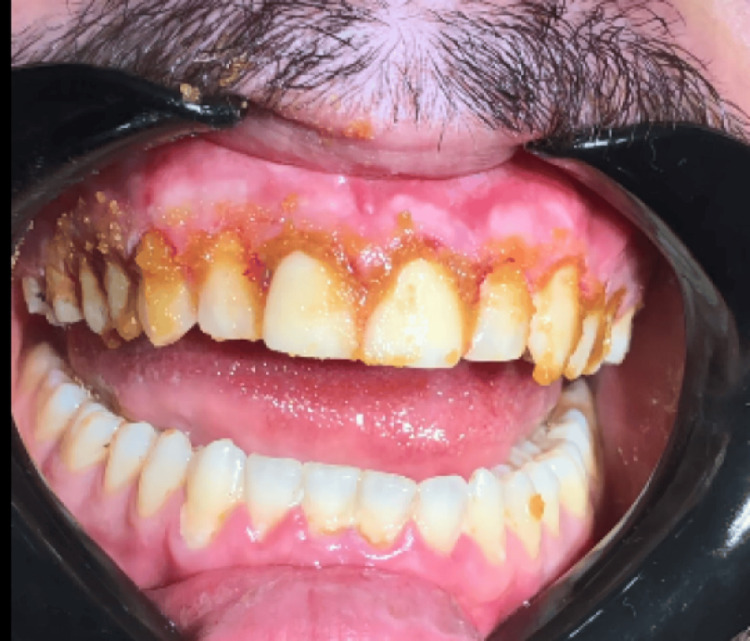
The topical administration of the medicament onto the gingival tissues

After completing the gingival trimming procedure, Orazor Oral Paste (Avenzor Pharmaceuticals) was applied to the treated area (Figure 4).

**Figure 5:**
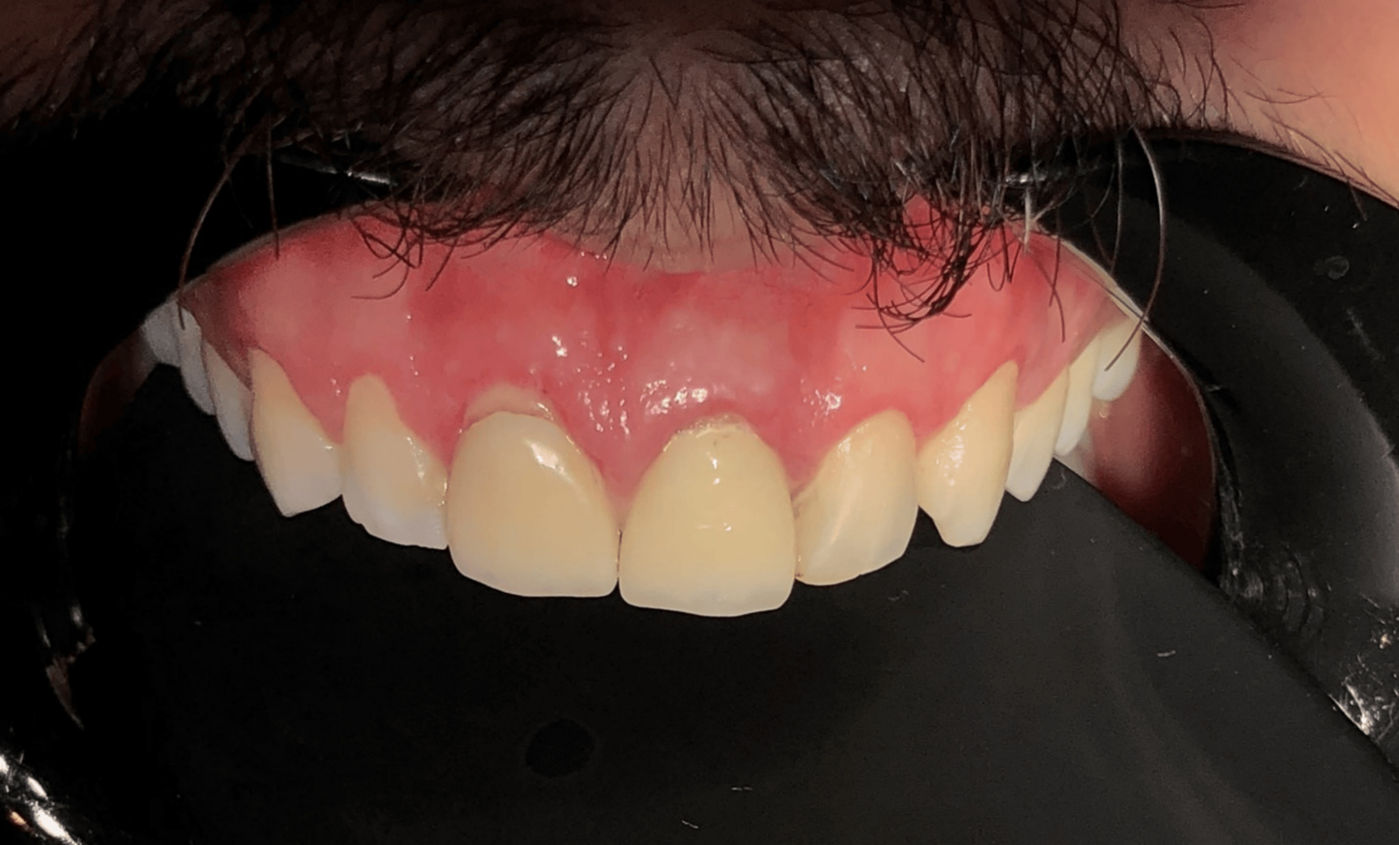
Gingival healing was observed seven days post-procedure, following surgical gingival trimming and the establishment of appropriate tooth length, demonstrating reduced inflammation and restoration of tissue architecture Clinical presentation of gingival healing seven days following surgical gingival trimming and crown lengthening procedure, demonstrating marked reduction in inflammation and reestablishment of gingival tissue architecture. Notably, gingival recession is observed on teeth #11 and #21, both restored with preexisting crowns.

This topical application was intended to promote healing, provide antimicrobial protection, and reduce postoperative discomfort. The paste’s formulation facilitates adhesion to the mucosal surface, ensuring prolonged contact and effectiveness. The patient was instructed to maintain proper oral hygiene and was checked daily for seven consecutive days to monitor healing progress and detect any potential adverse reactions. Follow-up evaluations indicated satisfactory healing without complications, highlighting the efficacy of Orazor Oral Paste in supporting tissue recovery post-gingival trimming (Figure 5).

**Figure 6:**
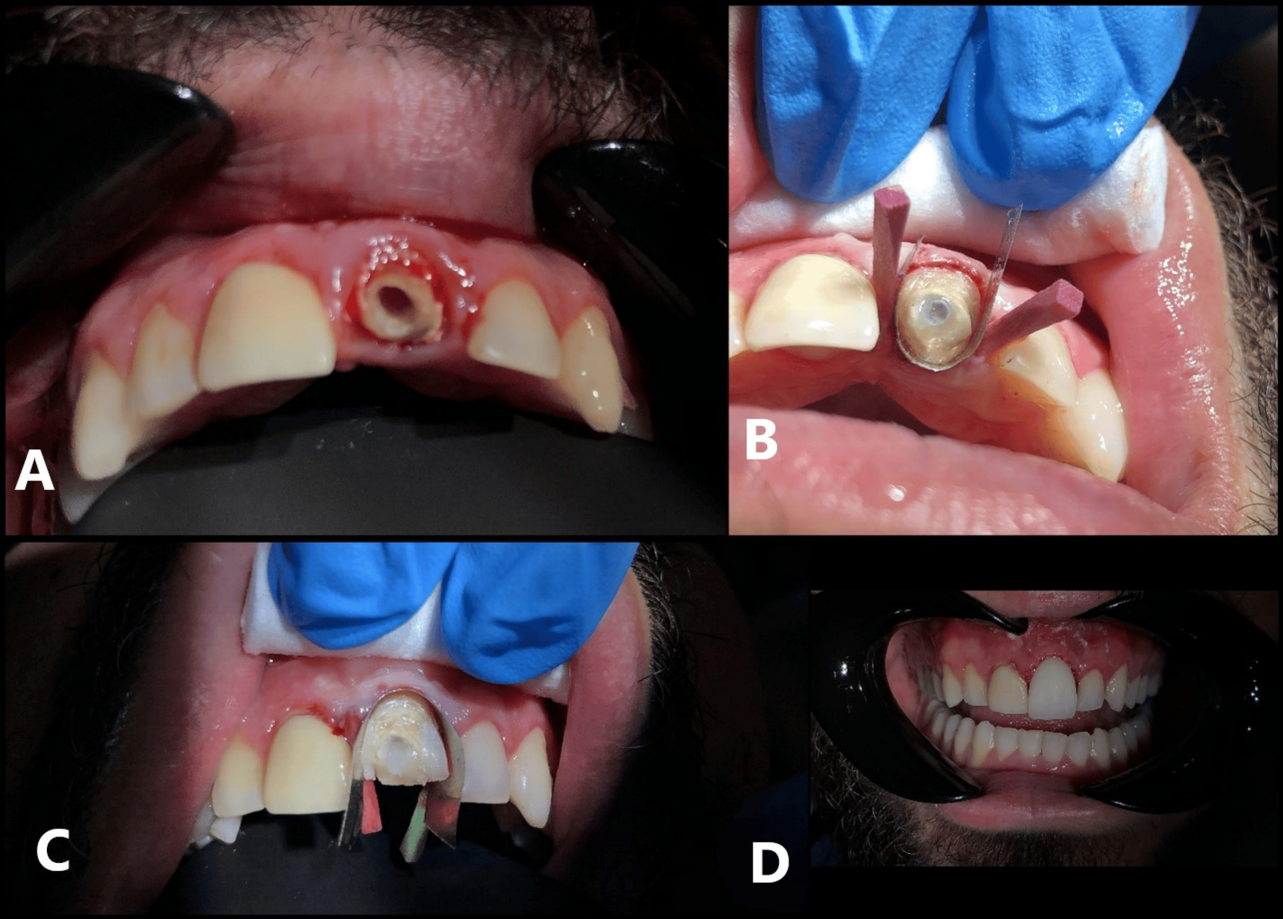
Stepwise clinical procedure demonstrating the restoration of tooth #21 with a direct composite resin crown. A. Tooth #21 following crown removal, exhibiting extensive structural damage. B. Application of the crescent metal matrix from the lingual aspect and placement of the fiber post to reinforce the root structure. C. Placement of the crescent metal matrix from the buccal aspect to establish proper esthetic contours and marginal adaptation. D. Final restoration of tooth #21 with a direct composite resin crown, demonstrating anatomical form and surface finish.

The most challenging aspects of the case involved managing teeth #21 and #11, both of which had pre-existing crowns (Figure 5). Upon removal of the crown on tooth #21, extensive destruction of the tooth structure was revealed. Fortunately, the endodontic treatment was satisfactory, providing a stable foundation for restorative rehabilitation (Figure 6A).

After discussing the treatment options with the patient, it was decided to rebuild both teeth #21 and #11 using customized composite crowns to improve their esthetic appearance and function.

The restoration process for tooth #21 included reinforcement with a fiber post, followed by the fabrication of a fully customized, handmade composite crown. The Crescent Metal Matrix was essential during this procedure, initially applied from the lingual aspect to establish proper contour and support, and subsequently from the buccal side to refine the esthetic form and margins. This stepwise technique allowed for precise shaping, excellent adaptation, and optimal esthetic integration (Figures 6B-6D).

**Figure 7:**
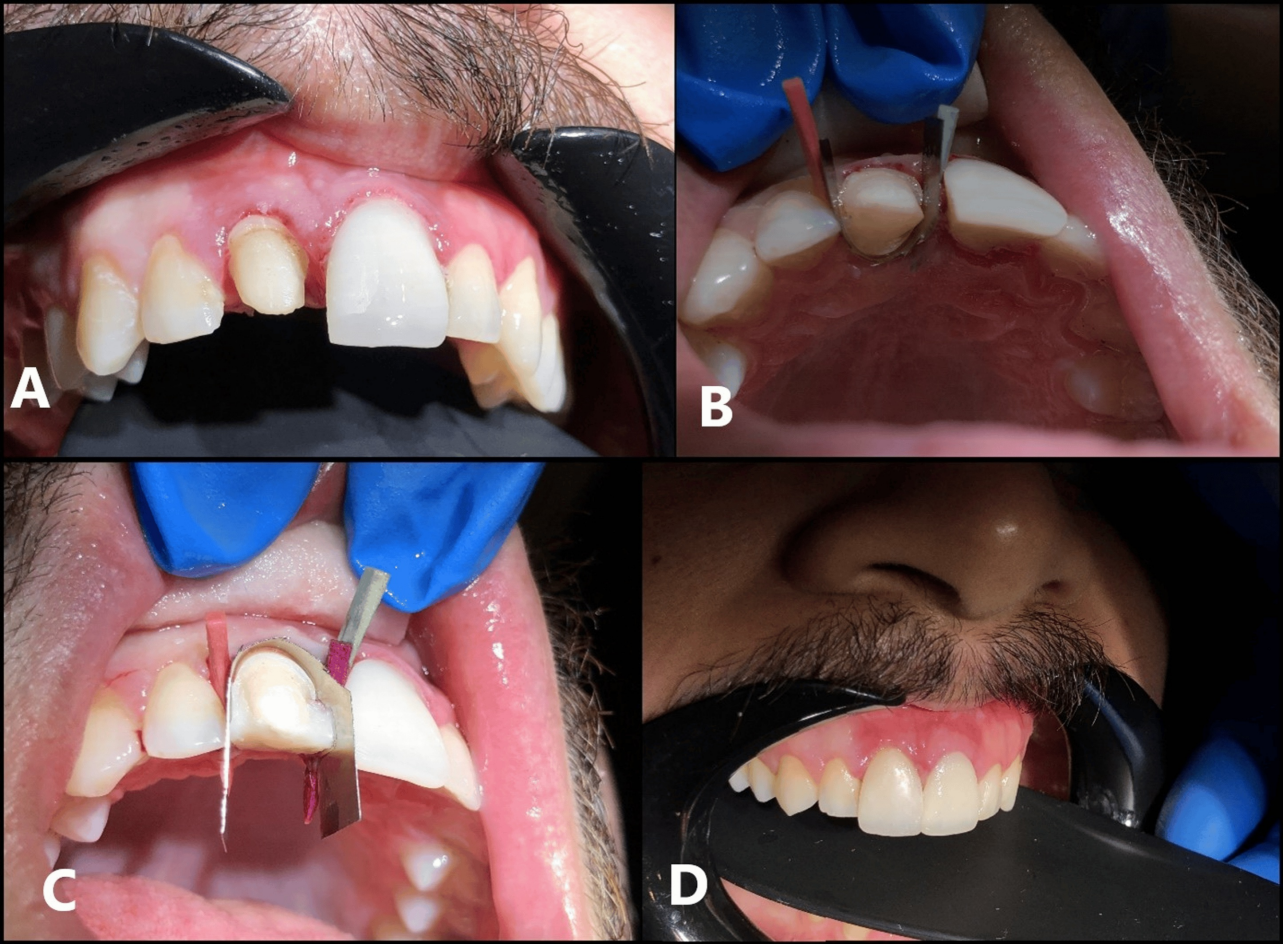
Sequential steps in the restoration of tooth #11 using a direct composite resin crown A. Tooth #11 after crown removal, displaying minimal remaining tooth structure. B. Application of the matrix band from the lingual aspect to facilitate restoration. C. Placement of the matrix band from the buccal aspect to ensure proper contour and marginal adaptation. D. Final restoration with a composite crown, demonstrating symmetry and consistency with the contralateral anterior restoration.

Following the successful restoration of tooth #21, the same technique was employed to rehabilitate tooth #11, ensuring harmony, symmetry, and consistency between the two anterior restorations (Figures 7A-7D).

**Figure 8:**
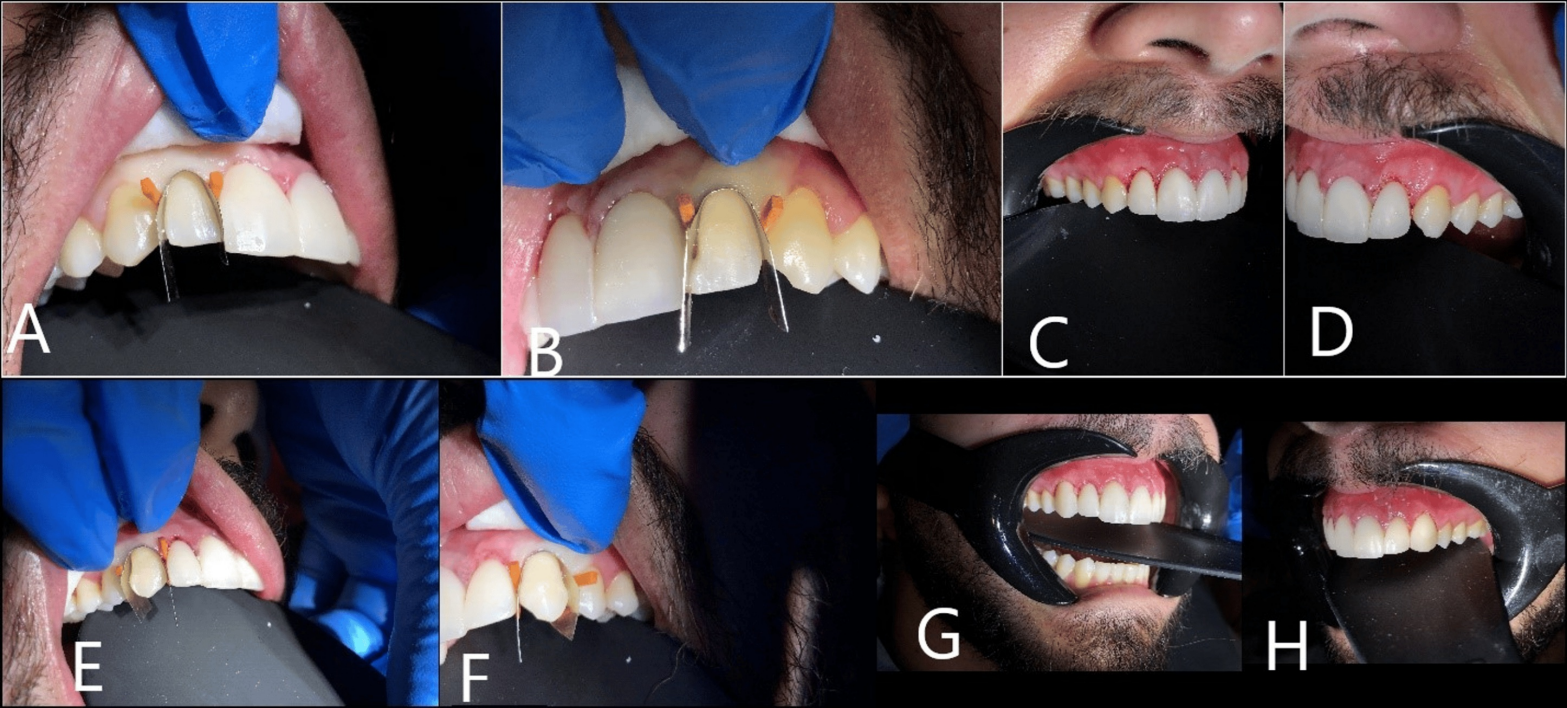
Sequential application of the anterior matrix system and direct composite resin veneers on maxillary anterior teeth (A and B) Application of the crescent metal matrix on teeth #12 and #22, respectively. (C and D) Illustrate the completed composite resin veneers on teeth #12 and #22, respectively, highlighting the final esthetic outcomes. (E and F) Application of the crescent metal matrix on teeth #13 and #23, respectively. (G and H) Illustrate the completed composite resin veneers on teeth #13 and #23, respectively, highlighting the final esthetic outcomes.

Following the precise gingival trimming and the restoration of teeth #21 and #11 with composite crowns, composite veneers were applied using the Crescent Metal Matrix, which played a crucial role in contouring and defining the optimal margins for each tooth (Figure 8).

**Figure 9:**
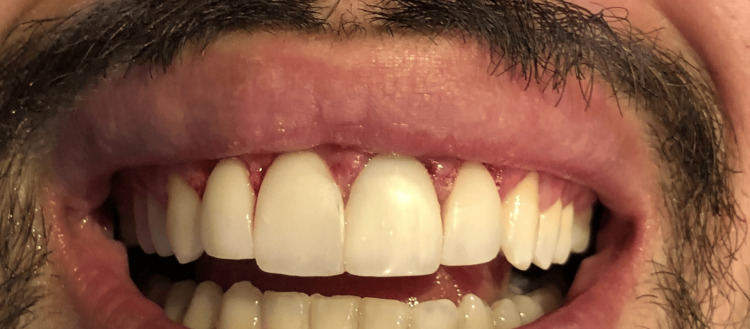
The patient's smile immediately following the application of direct composite resin veneers on the maxillary anterior teeth, demonstrating the immediate esthetic enhancement achieved through this minimally invasive procedure After applying the composite, the gingiva was gently reshaped using a soft tissue trimmer along with the matrix in place to ensure proper adaptation and positioning around the veneers, which facilitated accurate recording of the restoration margins.

Thanks to its pre-contoured design, the matrix closely conformed to the natural tooth anatomy, allowing for accurate reconstruction of the cervical and proximal contours. This enabled precise placement of the composite material, creating well-defined margins and guiding the careful repositioning of gingival tissue. After the composite application, the gingiva was gently retrimmed to ensure proper adaptation and relocation around the veneers, facilitating accurate recording of the restoration margins. The use of the Crescent Metal Matrix provided controlled shaping of the veneers, promoting seamless integration with the gingiva and contributing to excellent esthetic and periodontal outcomes (Figure 9).

Follow-up and outcome

**Figure 10:**
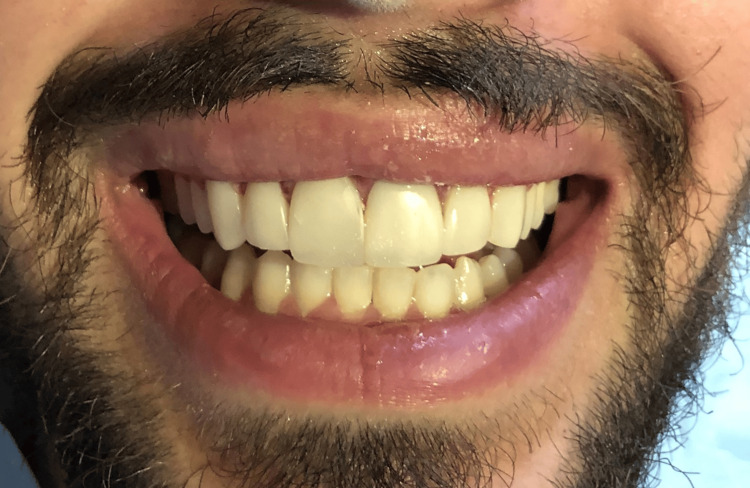
Displays the follow-up image taken three months post-treatment The gingival tissues demonstrated a healthy and stable condition, with no evidence of inflammatory response.

Follow-up assessments were scheduled at one week, one month, three months, and 12 months to monitor healing, periodontal stability, and esthetic outcomes. Patient-reported outcomes were recorded at each visit to capture subjective satisfaction and comfort, following protocols recommended in recent literature [8]. At the three-month appointment, the gingival tissues appeared healthy and stable, showing no signs of inflammation. The composite restorations were well-integrated with the surrounding teeth and soft tissues. The patient reported no sensitivity or discomfort and expressed high satisfaction with both the improved esthetics and function. Overall, the periodontal health was preserved, indicating a successful outcome of the treatment plan at this stage (Figure 10).

## Discussion

This case highlights a multifaceted approach to managing a young adult patient presenting with excessive gingival display (gummy smile) and esthetic concerns related to small anterior teeth, complicated by pre-existing restorations on teeth #11 and #21. The successful treatment outcome underscores the importance of a comprehensive assessment and meticulous planning when addressing esthetic and periodontal challenges concurrently.

Excessive gingival display during smiling can result from a variety of etiologies, including altered passive eruption, vertical maxillary excess, or gingival hyperplasia, often requiring a multidisciplinary treatment approach [9]. In this case, controlled gingival recontouring was essential to improve the smile line and restore harmonious tooth proportions. Preservation of the biological width during gingival trimming is critical to maintain periodontal health and prevent future recession or inflammation [10]. The innovative use of the Crescent Metal Matrix as a gingival retraction and trimming guide facilitated precise and conservative tissue removal, which is a significant advantage over traditional freehand techniques.

Orazor Oral Paste (Avenzor Pharmaceuticals) contains triamcinolone acetonide 1 mg per gram as its active ingredient, a potent corticosteroid known for its anti-inflammatory and immunosuppressive properties. This topical paste is formulated to reduce inflammation, alleviate pain, and promote healing in oral mucosal tissues. Its adhesive nature ensures prolonged contact with the affected site, enhancing therapeutic efficacy. Clinical use of Orazor has shown favorable outcomes in managing postoperative oral disorders by minimizing discomfort and accelerating tissue recovery [11].

Restoration of teeth #11 and #21 was particularly challenging due to the presence of pre-existing crowns and significant tooth structure loss, especially in tooth #21. The decision to rebuild tooth #21 using a fiber post and a customized handmade composite crown allowed for reinforcement and preservation of the remaining tooth structure, aligning with minimally invasive restorative principles. Tooth #11 was similarly restored with a customized composite crown to harmonize esthetics within the anterior segment. The Crescent Metal Matrix again proved instrumental during these restorative phases by facilitating accurate anatomical contouring and margin definition, critical factors for esthetic success and periodontal health [12].

Follow-up assessments demonstrated stable gingival health and structural integrity of the restorations at one week, one month, and notably at one year. Long-term preservation of periodontal health alongside esthetic success supports the effectiveness of combining innovative tools and materials with established clinical protocols. This case reinforces the value of personalized treatment planning and the integration of novel adjuncts in complex esthetic rehabilitations.

Limitations of this report include the single-patient nature and the need for further clinical research to validate the long-term benefits of the Crescent Metal Matrix. Nonetheless, this case contributes valuable clinical insights into managing gummy smiles and compromised anterior teeth esthetics with conservative and precise techniques.

While this report is largely descriptive and lacks quantitative periodontal measurements, steps were taken to transparently report clinical outcomes and any adverse events, in line with recommended guidelines for reporting harms in surgical and acceleration techniques [13].

## Conclusions

This case illustrates that a carefully planned, multidisciplinary approach combining precise gingival contouring and customized restorative techniques can effectively improve esthetic outcomes in patients with excessive gingival display and compromised anterior teeth. The use of tools such as the Crescent Metal Matrix, along with advanced restorative materials and techniques, contributed to favorable functional and esthetic results while maintaining periodontal health. However, as a single-case report with limited objective measurements and follow-up, the long-term stability of these outcomes cannot be definitively confirmed. Further studies with standardized, quantitative assessments and extended follow-up are warranted to validate the reproducibility and durability of this approach.
